# The Modern Treatment of Syphilitic Diseases, Both Primary and Secondary. Comprising Numerous Formulæ for the Preparation and Mode of Administration of the New Remedies; and an Account of a Safe and Successful Mode of Treating Chronic, Protracted, and Constitutional Syphilis, by the Mercurial Vapour-Bath

**Published:** 1846-04

**Authors:** 


ART. III.—*The Modern Treatment of Syphilitic Diseases, both Primary and Secondary. Comprising numerous formulæ for the preparation and mode of administration of the new remedies; and an account of a safe and successful mode of treating chronic, protracted, and constitutional syphilis, by the mercurial vapour-bath.* By LANGSTON PARKER, Surgeon to the Queen's Hospital, Birmingham, &c., &c. *Second Edition.—London,* 1845. 8vo. pp. 228.

WE noticed the first edition of this book on two former occasions (Vol. IX., p. 239, Vol. X., p. 381), and gave a very favorable report of its contents. The present edition is considerably enlarged and much improved. It is, indeed, entirely rewritten. The following extract from the preface indicates the character of the principal changes made in the work.

"Six years' additional experience, both in hospital and private practice, has enabled me to confirm the efficacy of most of the plans of treatment recommended in the first edition of this work. Much new matter has been added in the present edition, chiefly, if not altogether, original. That which I regard of the first importance, is the account of the treatment of various forms of syphilis by the mercurial vapour-bath. Diseases rebellious or tedious under ordinary treatments, generally yield with ease to this combination, more particularly affections of the skin and bones; the duration of treatment is by it much shortened, the quantity of medicine required to be given by the mouth greatly diminished, and the cures rendered more permanent and certain. Relapses after this mode of treatment I have found extremely rare; whereas under the ordinary plan they are exceedingly frequent, even after a perfect cure had been supposed to have been effected."

We strongly recommend this volume to the attention of our practical readers. It is the only recent complete manual we possess, of a moderate size, on the subjects of which it treats, and ought to be in the possession of every young surgeon who is called on to treat venereal affections.

